# Reproductive rights and options available to women infected with HIV in Ghana: perspectives of service providers from three Ghanaian health facilities

**DOI:** 10.1186/1472-6874-13-13

**Published:** 2013-03-15

**Authors:** Amos Kankponang Laar

**Affiliations:** 1 Department of Population Family and Reproductive Health, School of Public Health, College of Health Sciences, University of Ghana, Legon, Box LG 13, Accra, Ghana

## Abstract

**Background:**

Owing to improved management of HIV and its associated opportunistic infections, many HIV-positive persons of reproductive age are choosing to exercise their right of parenthood. This study explored the knowledge of health workers from two Ghanaian districts on the reproductive rights and options available to HIV-positive women who wish to conceive.

**Methods:**

Facility-based cross-sectional in design, the study involved the entire population of nurse counselors (32) and medical officers (3) who provide counseling and testing services to clients infected with HIV. Both structured and in-depth interviews were conducted after informed consent.

**Results:**

Two main perspectives were revealed. There was an overwhelmingly high level of approbation by the providers on HIV-positive women’s right to reproduction (94.3%). At the same time, the providers demonstrated a lack of knowledge regarding the various reproductive options available to women infected with HIV. Site of facility, and being younger were associated with practices that violated client’s right to contraceptive counseling (p < 0.05) in each case. Some of the providers openly expressed their inability to give qualified guidance to HIV-positive women on the various reproductive options.

**Conclusions:**

Taken together, these findings suggest that many HIV-positive clients do not receive comprehensive information about their reproductive options. These findings highlight some of the problems that service providers face as HIV counselors. Both service providers and policy makers need to recognize these realities and incorporate reproductive health issues of HIV-persons into the existing guidelines.

## Background

Prior to the widespread availability of antiretroviral therapy (ART), the desire to conceive among HIV-infected women was discouraged due to a high perinatal transmission risk [1]. The recent advancement in HIV therapy and improved treatment of its associated opportunistic infections has transformed the conceptualization of AIDS from a prospect of death to one of management of a chronic illness. Perinatal transmission now ranges from 0% to 5% with appropriate use of antiretroviral drugs during pregnancy, delivery, and in the newborn infant [2,3]. As a result, the proportion of HIV-positive individuals of reproductive age who desire to exercise their fundamental right to reproduction is increasing.

It is worth noting that HIV-positive persons like non-infected persons are entitled to all the rights as enunciated in articles 1 through 30 of the Universal Declaration of Human Rights [4]. These include rights to marriage and reproduction. Mindful, however, of perinatal transmission of HIV and related risks, the World Health Organization (WHO), the Joint United Nations Program on HIV and AIDS (UNAIDS) and other partners in 2004 came out with a 4-pronged strategy for HIV and pregnancy [5]. This strategy entails “preventing HIV infection in young people and women of childbearing age; preventing unwanted pregnancy among women with HIV infection; preventing transmission of HIV from an infected mother to her infant and support for HIV infected women, infants and families”.

Following this, educational interventions have focused on the first strategy [6]. These interventions though efficacious have not been very effective (large numbers of youth and women worldwide continue to contract HIV) due to factors beyond their control. In comparison, measures instituted toward the third strategy (preventing perinatal transmission) have proved very effective [7,8]. Prevention of mother-to-child transmission (PMTCT) as it is called has thus become a major element of many governmental HIV programs. An unfortunate consequence of the focus on PMTCT is that women have been approached mainly as vectors of HIV transmission. In some instances, women with HIV are told that they should not have children or they should take antiretroviral drugs (ARVs) to prevent infection of their babies [9].

With these rights and strategies come options for HIV-positive persons. Herein described are the various reproductive options. It is important to note that among seroconcordant couples (that is both individuals are infected with HIV), conception can still occur naturally through unprotected intercourse. The additional risk of transmitting drug-resistant HIV to the other partner in a process known as HIV superinfection [10,11]. This can be minimized if both partners are receiving ART, have an undetectable viral load and carefully time the unprotected intercourse at the midcycle, when the woman is most fertile.

In the case of serodiscordant couples (where the woman is HIV-positive and the male is negative or vice versa), there is the risk of sexually transmitting the virus to the other partner during unprotected intercourse. Research has shown that having an undetectable viral load in the blood does not eliminate the possibility of transmitting the virus as levels of HIV in the blood and semen or vaginal secretions are poorly correlated [12]. On average, the risk per unprotected coital act (unprotected vaginal intercourse) is estimated to be between 0.05 to 0.15% for females and 0.03% to 0.09% for males [12].

Another option to consider is washed intrauterine insemination (IUI), commonly referred to as artificial insemination. IUI eliminates the need for intercourse and subsequent risk of HIV transmission. In this case special techniques are used to separate the sperm from the seminal fluid. The sperms are then placed directly into the uterus after accurately assessing the time of ovulation. The pregnancy rate per cycle of IUI is 10% to 12% [13]. Another option available to couples in whom the male is HIV-positive and the woman is negative is insemination using donor sperm.

A commonly performed procedure in developed countries is in vitro fertilization. The specific technique used is called intracytoplasmic sperm injection whereby the woman's oocytes (eggs) are harvested after undergoing ovulation induction and the male's sperm is isolated. The technique involves fertilizing each egg with one sperm instead of exposing the egg to millions of sperm as is done in IUI. Once several eggs have developed into embryos, they are then transferred back to the uterus and the woman is monitored using a blood pregnancy test to determine if implantation was successful.

Over the past decade, adjunctive therapies, such as elective Caesarean section, Vitamin supplementation and antiseptic washes have been suggested for adoption by HIV-positive women [14]. One argument for the elective Caesarean section procedure is its potential value in preventing or reducing perinatal HIV transmission during the birthing process. One such evidence was unveiled in 2001 by The International Perinatal HIV Group. They showed that, the risk of perinatal HIV transmission increases by 2% with every 1-hour increase in the duration of membrane rupture before delivery in HIV-infected women regardless of whether women receive a mono or combination antiretroviral therapy [15]. Other authorities have, however, raised counter arguments that women infected with HIV might suffer increased morbidity and mortality with Caesarean sections such as delayed wound healing, high fevers, severe anemia, infections and a need for additional surgery [16]. A review that included one randomized clinical trial which assessed the efficacy of elective Caesarean section delivery for the prevention of mother-to-child transmission of HIV, three cohort studies, one secondary data analysis from a clinical trial and one case–control study [17] showed that the incidence of postpartum morbidity and mortality was higher among HIV-infected pregnant women who had delivered by Caesarean section compared with those who had delivered vaginally. The review concluded that women should be informed about the risks associated with Caesarean section delivery along with the potential benefits expected from this procedure [17]. A related review [18] while acknowledging the utility of the procedure for preventing vertical transmission in the absence of complete viral suppression, also warn of the potential health risks of the procedure to mothers and babies owing to a lack of technical expertise, the availability of adequate aseptic conditions, or both, as well as cost. These reservations are particularly relevant in resource-limited settings.

Adoption is yet another option for those couples who do not want to risk HIV transmission from unprotected intercourse or who fail or choose not to undergo assisted reproductive techniques [19].

While the various options described above are available in Ghana, their prohibitive costs make them unattractive to this group of clients under discussion as well as the general Ghanaian public (Dr. Maxwell Antwi, La General Hospital; Personal Communications). The costs range from six hundred Ghana cedis (approximately 400 US dollars for elective Caesarean section) to seven thousand Ghana cedis (approximately 4,600 US dollars for IUI).

With this reality of a widening spectrum of reproductive choices for HIV-infected persons, experts advise that health professionals not only look at the disease as an entity in itself but to approach the HIV-positive client as a person taking his/her right to a reproductive life into consideration [20,21]. As to whether the Ghanaian healthcare provider is abreast of this reality was one of the questions that motivated this inquiry. The study explored the perspectives of counseling and testing (CT) as well as PMTCT service providers on the reproductive rights and options available to HIV-positive women who wish to conceive. It also determined whether site of facility and some selected demographic attributes of the providers were related to contraceptive prescription to HIV-positive clients. It further assessed the in-depth knowledge (on reproductive matters of persons infected with HIV) of a subgroup of service providers unto which the management of the PMTCT Programs was entrusted. The data generated will help strengthen the existing service provision guidelines and practices.

## Methods

### Study design, study sites and population

This study was facility-based cross-sectional in design involving 32 nurse counselors/PMTCT service providers, and three medical officers from the Tema General Hospital (Tema, Accra, Ghana), the Atua Government Hospital, and the St Martin’s Hospital (both in the Manya Krobo District, Eastern Region, Ghana). All the three study hospitals provide PMTCT services such as CT, infant feeding counseling and ART to both rural and urban populations. These 35 participants represent the entire population of health workers who were providing these services to HIV-infected clients at the hospitals.

The districts/hospitals were purposively selected on the basis of their high HIV prevalence and the existence of relevant structures on the control of the epidemic. For instance, the two sites in the Manya Krobo district were the first PMTCT pilot sites in Ghana. In this district, HIV prevalence among pregnant women in the district over the years have consistently been above the national average ranging from 18% in 1992 to 8.9% in 2007 [22]. According to the report of the district health directorate in 2006, the proportion of HIV-infected women accessing postnatal services at the district was 6.0% [23]. Service data from the Tema Municipal Health Directorate indicate identify HIV as a major health problem among pregnant women with a prevalence of 3.6% in 2006.

### Data collection tool design/adaptation, pre-test and validation

Two tools were employed in eliciting the perspectives of healthcare providers on the subject of reproductive rights of, and reproductive options for PLHIV. A structured questionnaire (tool 1) was used in carrying out structured interviews that involved the entire population (35 health workers) providing HIV-related services at the three hospitals. An in-depth interview guide (tool 2) was used to facilitate the in-depth interviews with all three service providers managing the PMTCT programs of the respective facilities.

### Tools design/adaptation

The adaptation process involved a diligent review of literature and existing tools. Relevant questions were culled from a number of the literature cited in this manuscript [1, 6, 16, 20 – 21, 25 – 27] and incorporated into the study’s draft questionnaire/guide. In particular, the current study tools benefited from those of Leshabari et al. [24], and de Bruyn [25]. It is the practice at the School of Public Health in Legon, to have faculty members peer-review both study proposals and draft tools before submitting to the University Institutional Review Board (IRB) for a second round of techinal and ethical reviews. This particular study was not an exception. The funders of this study (The Gates Institute of the Johns Hopkins Bloomberg School of Public Health, in Baltimore, USA), who are duly acknowledged in this manuscript also provided technical review of the tools and the protocol at the initial stages of the study.

### Tool pre-test, validation and general quality assurance measures

Prior to the data collection, the draft tools were pre-tested on midwives providing PMTCT services at the Ashaiman Health Center, a neighbouring district of the Tema Municipality. The validation process at this stage involved minor modifications of the draft tools to reflect cultural appropriateness issues identified during the pre-testing.

Additional safeguards instituted to improve data quality included such quality assurance measures as the author single-handedly collecting the data at all the three sites, and also managing both the data entry and analyses.

### Actual data collection

Knowledge of the 35 service providers on the reproductive matters of HIV-positive women was assessed through structured interviews. The tool used documented the demographic characteristics of as well as their perspectives on matters relating to reproductive rights and options for HIV-infected women. These included their acknowledgement of HIV-positive client’s rights to procreation, their awareness of reproductive options for HIV-positive clients, their willingness to prescribe unprotected intercourse to HIV-positive clients as an option for conception. It also assessed their level of awareness of adoption transactions by HIV-positive couple in their communities, and whether or not they will offer contraceptive services to HIV-positive client during their counseling sessions.

Additionally, in-depth interviews involving three of the counselors in charge of the PMTCT programs at the three hospitals were done. Through a semi-structured interview guide, the in-depth interviews elicited the providers’ perspectives on the reproductive rights, and reproductive options available to women infected with HIV. All interviews were conducted in English and recorded in writing.

### Ethical issues

The study protocol was first reviewed by the faculty members of the School of Public Health, University of Ghana, Legon for appropriateness and scientific content. An ethical clearance was afterwards obtained from the Institutional Review Board (IRB) of the Noguchi Memorial Institute for Medical Research, University of Ghana, Legon.

Written informed consent was obtained from all 35 service providers. They were informed about the objectives and methods of the study. They were also assured of strict confidentiality concerning any information they would give.

### Data analysis

Statistical analysis of the quantitative data was done using PASW Statistics 18, Release Version 18.0.0 [26]. Associations between certain demographic attributes of the providers and their behavior/level of knowledge were tested using the Chi Square test. Further evaluations of these associations were done using logistic regression technique, where odds ratios (ORs) and their 95% confidence intervals (CI) were computed.

The data from the in-depth interviews were analyzed manually. This consisted of appraising the jotted notes, and synthesizing them into meaningful narrative by the author. Certain portions of the participants’ responses were also reported verbatim where it was deemed necessary; others were given minor edits to enhance readability.

## Results

### Characteristics of service providers and their perspectives on reproductive rights and options available to women infected with HIV

The background characteristics of the service providers as well as their perspectives on reproductive rights and options are presented in Tables 1 and 2. The mean age of the providers was 31 years (range 20–60 years). They were all Christians and the majority were currently or previously married.

**Table 1:** Characteristics and perspectives of service providers on reproductive rights and options available to women infected with HIV

**Attribute**	**Frequency**	**Percent**
**Sex of provider**		
Male	2	5.7
Female	33	94.3
**Facility**		
Atua Government Hospital	9	25.7
St Martin’s Hospital	14	40.0
Tema General Hospital	12	34.3
**Age of Provider**^**1**^		
Under 30 years	14	40.0
Over 30 years	21	60.0
**Marital status of provider**		
Never married	14	40.0
Currently married	17	48.6
Ever married	4	11.4
**Religious affiliation**		
Christian	35	100.0
**Place of residence**		
Urban	18	51.4
Rural	17	48.6
**Level of education**		
Tertiary	22	62.9
Other	13	37.1

^1^ Mean age was 40, ranged from 20 – 60 years.

**Table 2:** Perspectives of service providers on reproductive rights and options available to women infected with HIV

**Questions exploring providers perspectives on reproductive matters of women infected with HIV**	**Response (Yes)**	**Proportion (%)**
Do people infected with HIV have the right to ‘have children’?	33	94.3
Are you aware of the various reproductive options^2^ for HIV-positive clients?	4	11.4
Will you prescribe unprotected intercourse to a person infected with HIV as an option for conception?	9	25.7
Are you aware of any adoption transaction by HIV-positive couple in your community?	1	2.9
Are you aware of any adoption services in this community?	4	11.4
Will you offer contraceptive services to HIV-positive client as part of your counseling session?	20	57.1
Are you aware of any safe measures that can help women infected with HIV to conceive and deliver safely?	8	22.9

^2^The only options mentioned were adoption and surrogacy.

The findings reveal two main perspectives regarding the knowledge of the service providers on the reproductive matters of women infected with HIV. There was an overwhelmingly high level of approbation by the providers on HIV-positive women’s right to reproduction (94.3%). A considerable proportion of the providers (57.1%) also asserted that women infected with HIV have the same rights as their uninfected counterparts to reproduction and contraception. These providers at the same time demonstrated a disappointingly high level of ignorance regarding the various reproductive options available to women infected with HIV in Ghana. Only eight (22.9%) of the providers were aware that measures exist to help HIV-positive women conceive and deliver safely: These measures mentioned were the use of antiretroviral drugs and delivery via elective Caesarean section. Four of the 35 (11.4%) were aware of reproductive options for HIV-positive women, and only 9 (25.7%) would advise HIV-positive women to have unprotected intercourse as an option to conceive. Some of the providers openly expressed during the interactive in-depth interviews their inability to give qualified and relevant advice to HIV-positive women on the various reproductive options. The constraints mentioned were lack of resources particularly guidelines, and refresher training to update their knowledge.

### Background of the participants of the in-depth interviews

Three in-depth interviews were conducted with the service providers with designations “PMTCT In-charges” at the three hospitals. All three were nurses with midwifery qualification as well as public health. They were all females and aged 45, 57 and 59. All of them, in addition to their administrative and managerial roles also provide PMTCT services to clients who visited their facilities. All three had recently received some training in PMTCT.

### Perspectives of service providers on reproductive matters of HIV-infected women

The findings of the in-depth interviews corroborated those of the quantitative study. They providers agree that HIV-positive women have the right to reproduction. The following narratives typify their experiences, their level of knowledge or awareness on these matters.

When asked whether they were aware of any guidelines on HIV and reproductive rights or options, none of them was sure of the existence of such a guideline. One of them said she had heard about it.

Another provider states:

‘If you are talking about specific guidelines on reproductive rights or options, I really don’t know what you are talking about. What we have is the one by WHO on Infant Feeding. We use this all the time, not reproductive rights….”

With regards to specific training on reproductive health issues of HIV-infected women, all the nurses stated that they did not receive any training as nurse trainees or on the job. This nurse further explains:

‘I completed midwifery school about 21 years, when we were in midwifery school HIV was not in the curriculum we didn’t hear much about the HIV’ (name of hospital withheld).

All they providers expressed their desire to have regular opportunities for training:

‘I want to be abreast with the latest issues, I want to go for more workshops. I don’t want to be stuck at the same place’

### Associations between some selected attributes of providers and willingness to offer contraceptive service to HIV-positive client

Further exploration of the data showed that a significantly higher proportion of providers from the Manya study site (χ^2^_(1)_ = 8.11; p = 0.004), and those younger than 30 years (χ^2^_(1)_ = 4.47; p = 0.035), found it inappropriate to provide contraception to HIV-positive clients. Compared to their counterparts from the Manya study site, providers from the Tema site were more likely to prescribe to their clients unprotected intercourse as a reproductive option OR = 2.56; 95% CI (1.11 – 5.85). So were providers with tertiary level education OR = 1.86; 95% CI (1.14 – 3.02). In a multiple logistic regression analysis that controlled for sex of the provider, the predictive ability of a provider’s age though attenuated retained its statistical significance (Table 3).

**Table 3:** Predictors of contraceptive prescription to HIV-positive clients

**Predictors**	**OR**^**1**^	**95% CI**^**1**^	**OR**^**2**^	**95% CI**^**2**^
**Site of Provider**				
Tema	2.56	1.11 – 5.85	1.99	0.23 - 16.86
Manya Krobo				
**Level of education**				
Has tertiary level qualification	1.86	1.14 – 3.02	2.45	0.39 – 15.38
Education below tertiary level				
**Age of provider**				
30 years or younger	0.19	0.04 – 0.94	0.20	0.05 – 0.95
Older than 30 years				

^1^Odds Ratio from simple logistic regression; ^1^95% CI for odds ratio; ^2^Odds Ratio from logistic regression that controlled for sex of provider; ^2^95% CI for odds ratio; Cox & Snell R Square = 0.137.

## Discussion

Anecdotes and local speculations have long hinted on the need to strengthen existing service provision guidelines and to equip service providers with the requisite competencies to interact with HIV-positive clients professionally. In the case of HIV, it is not only the medical facts that are relevant to their health, but also many of the psychosocial issues relevant to their interest in having children [6]. Health workers themselves have sent out several calls for knowledge updates through refresher training. One does not have to carefully peruse the yearly reports churned out by the Ghana Health Services to recognize this. This current study on providers’ awareness and in-depth knowledge on reproductive matters of persons infected with HIV reveal two main perspectives. An overwhelming majority of the providers acknowledge HIV-positive person’s right to reproduction. However, these providers, at the same time, were very ignorant about the various reproductive options available to persons infected with HIV.

First, it was refreshing to note that nine out of every ten providers acknowledge HIV-positive person’s right to reproduction. This is in line with articles 1 and 16 of the Universal Declarations of Human Rights, which state that *“All human beings are born free and equal in dignity and rights…”;* and that “*men and women of full age, without any limitation, have the right to marry and to found a family…”.*

However, when the in-depth knowledge of these providers was scrutinized, only 1 in five were aware that measures exist to help HIV-positive women conceive and deliver safely. The measures mentioned were the use of antiretroviral drugs and delivery via elective Caesarean section. de Bruyn [6] notes of a gathering in 2004, where more than 25 national and international organizations presented a statement to the secretariat of the United Nations Commission on the Status of Women that highlighted relatively neglected areas in the reproductive health of women affected by HIV and AIDS [25]. Ipas in collaboration with the International Community of Women Living with HIV, the Center for Health and Gender Equity and the Pacific Institute for Women’s Health, used that statement and a literature review [6] to develop a practical tool to help interested organizations address those neglected areas of reproductive health. Delineated in this tool are various reproductive options. These have been described in detail in the introductory section of this paper.

A discussion on the perspectives and in-depth knowledge of the service providers needs to be nuanced. A careful scrutiny of the various guiding instruments used by the providers reveal no such guidelines on reproductive rights or options for HIV-positive clients. Secondly, while the various options described above are available in developed countries, they are usually unavailable in resource-poor settings. In places where these are available, they may not be affordable, or acceptable. For instance, while the various options described above are available in Ghana, their prohibitive costs make them unattractive to the group of clients under discussion as well as the general Ghanaian public (Dr. Maxwell Antwi, La General Hospital; Personal Communications). The costs range from six hundred Ghana cedis (approximately 400 us dollars for elective Caesarean section) to seven thousand Ghana cedis (approximately 4,600 us dollars for IUI).

Even if these procedures/options are affordable, there are other social, religious or moral concerns that preclude their adoption, For example, insemination with a donor sperm though perfectly safe, may not be acceptable due to the removal of possibility of genetic parenthood. This has tremendous moral and ethical implications. Adoption, depending on the setting may not be acceptable due to the presence of HIV infection in one or both partners. As such, these realities, and not only the appreciation of the medical risks involved, influence decision-making by service providers. Finally, given that there are no specific policies with regard to reproductive options for HIV persons accessing services in Ghanaian hospitals, it is not surprising that most of the health workers were ignorant about them.

A fundamental premise for successful counseling is that the counselor has both confidence in his/her own professional knowledge, and the relevant application of this knowledge for the individual client being counseled. About 1 in four of the providers would advise HIV-positive woman to have an unprotected intercourse as an option to conceive. Some of the providers openly expressed during the interactive in-depth interviews their inability to give qualified advice to HIV-positive clients on the various reproductive options. The constraints mentioned both in the structured and in-depth interviews were lack of resources particularly guidelines, and refresher training which are essential for knowledge update. This finding compares favorably with findings of previous investigations [24,27,28]. In their study Leshabari et al. explored the experiences and situated concerns of nurses in Tanzania. It revealed a high level of stress, frustration, and acknowledgment of incompetence by the nurse-counselors.

Could these perspectives be influenced by some demographic attributes of the providers? The designs of the studies cited precluded the exploration of this possibility statistically. The current analysis did. At the bivariate level, age, level of education, and site of provider were important. Significantly higher proportion of providers from the Manya study site, and those younger than 30 years, found it inappropriate to provide contraception counseling to HIV-positive clients (Table 3). With regard to age and education, it would seem natural that the younger and less educated/experienced providers would be challenged. Although not significant at the multivariate level, a higher proportion of providers without tertiary level education tended to withhold contraceptive information from their clients.

Taken together, the current findings suggest that HIV-positive clients do not receive comprehensive information about their reproductive options. Even though this may be motivated by legitimate concerns, such practices by the counselors infringe on the reproductive rights of HIV-positive clients. An alternative counseling approach that respects clients’ rights to informed and considered decision-making concerning childbearing should be encouraged. Such an approach would require contextualizing the counseling encounter with candid discussions. Unfortunately, this is not possible without the revising and introducing of reproductive health matters into the current service provision guidelines.

At this point, it is apropos to discuss a number of limitations that this study may suffer from. First, given the design of the study, these findings may not be generalized to the entire population of health workers providing these services in Ghana. It is, however, reasonable that the results, which are based not on one, but on three facilities, and which are collected through a triangulation of two methods, have considerable relevance for HIV-related service provision well beyond the three health facilities. Furthermore, the scope of the study is limited; only service providers are included. A more comprehensive exploration of problems that may compromise counseling on HIV and reproductive options would involve other groups of study participants. Views of HIV-positive women would be particularly relevant in this. To this end, a related study that explored the challenges that health workers face implementing PMTCT counseling, as well as the experiences of HIV-positive clients receiving these services at seven health facilities in the Greater Accra Region of Ghana show that providers face various challenges including lack of counseling acumen, inadequate logistics, inadequate training, and uncertainty about the credibility of counseling information [29]. The challenges presented in this paper may therefore not be unique to only the service providers of the three facilities, but may be experienced by other in various parts of Ghana.

## Conclusions

These findings bring to the fore the perspectives of services providers on reproductive health matters of women infected with HIV, as well as the challenges they face as HIV counselors. Recognizing these realities and incorporating reproductive health issues of HIV-positive persons into existing policies and guidelines as well as periodic knowledge upgrade through refresher training could improve service provision to both HIV-infected clients in Ghana.

## Competing interest

No conflict of interest whatsoever.

## Authors' contributions

The author solely conceived and designed the study. He managed the data collection process, and did the analysis and interpretation of data. He also singlehandedly drafted and finalized the manuscript.

## Pre-publication history

The pre-publication history for this paper can be accessed here:

http://www.biomedcentral.com/1472-6874/13/13/prepub
